# The Role of DNA Restriction-Modification Systems in the Biology of *Bacillus anthracis*

**DOI:** 10.3389/fmicb.2016.00011

**Published:** 2016-01-22

**Authors:** Ramakrishnan Sitaraman

**Affiliations:** Department of Biotechnology, TERI UniversityNew Delhi, India

**Keywords:** *Bacillus anthracis*, restriction enzymes, DNA methyltransferase, R–M, selfish genes, type IV restriction enzymes, methylation-dependent restriction enzyme, orphan DNA methyltransferase

## Abstract

Restriction–modification (R–M) systems are widespread among prokaryotes and, depending on their type, may be viewed as selfish genetic elements that persist as toxin–antitoxin modules, or as cellular defense systems against phage infection that confer a selective advantage to the host bacterium. Studies in the last decade have made it amply clear that these two options do not exhaust the list of possible biological roles for R–M systems. Their presence in a cell may also have a bearing on other processes such as horizontal gene transfer and gene regulation. From genome sequencing and experimental data, we know that *Bacillus anthracis* encodes at least three methylation-dependent (typeIV) restriction endonucleases (RE), and an orphan DNA methyltransferase. In this article, we first present an outline of our current knowledge of R–M systems in *B. anthracis*. Based on available DNA sequence data, and on our current understanding of the functions of similar genes in other systems, we conclude with hypotheses on the possible roles of the three REs and the orphan DNA methyltransferase.

## Introduction

The existence of restriction–modification (R–M) systems was initially inferred from the differential ability of bacteriophages to multiply on different strains of *Escherichia coli* (Arber and Dussoix, 1962; Arber et al., 1963). Since then, R–M systems have been found among an overwhelming majority of prokaryotes studied to date, both bacteria and archaea, and are classified into one of four types (recently reviewed by Loenen et al., 2014). **Table 1** lists some salient features of R–M systems that are relevant for this article. The simplest type of R–M system (now termed type II), consists of a restriction endonuclease (RE) and a cognate DNA adenine (*N*^6^) or cytosine (C-4 or C-5) methyltransferase (MTase), both of which are single, independently functioning polypeptide chains. Both REs and MTases are sequence-specific DNA binding proteins, having the same target sequence, but performing different actions upon recognition of these sequences. The RE degrades DNA molecules possessing unmethylated target sequences, while the MTase methylates the same target sequences (whether unmethylated or hemi-methylated), shielding them from RE activity. This acts as a defense mechanism against bacteriophage infection that is, in most cases, able to destroy viral DNA that is more likely to be unmethylated (or bearing an incompatible methylation pattern) as compared to the host DNA (reviewed by Pingoud et al., 2005). On the other hand it may be thought of as a ‘selfish’ toxin–antitoxin module, with residual RE molecules in the cytoplasm causing post-segregational killing upon loss of the R–M genes (Naito et al., 1995). Type I and III R–M systems are more complex than type II in terms of molecular architecture, but again essentially consist of cognate and antagonistic RE and MTase activities (reviewed by Bourniquel and Bickle, 2002). A fourth type (type IV) – methylation-dependent restriction enzymes (MDREs) – are REs that preferentially target modified DNA containing glycosylated bases, or methylated on adenine or cytosine residues, but lack a cognate MTase altogether. The lack of methylation may be considered to be the ‘modified’ state (recently reviewed by Loenen and Raleigh, 2014).

**Table 1:** Some salient features of R–M enzymes (Based on Bickle and Kruger, 1993; Loenen et al., 2014).

R–M Type	MTase composition	RE composition	Mode of function
I	Complex consisting of one sequence-specific DNA-binding subunit and two MTase subunits.	Complex consisting of one sequence-specific DNA-binding subunit, two MTase and two RE subunits.	Hemimethylated DNA is preferentially methylated relative to unmethylated DNA. ATP-powered translocation of unmethylated DNA precedes double-strand cleavage at random and distant sites from the initial binding site. Methylation and cleavage of DNA are mutually exclusive.
II	Single polypeptide chain with sequence-specific DNA binding and methylation activities.	Single polypeptide chain with sequence-specific DNA binding and cleavage activities. May or may not dimerize.	Methylation and cleavage of DNA are independent reactions. DNA cleavage occurs either within the recognition site, or sometimes at a fixed distance away from the site.
III	Single polypeptide chain that can carry out sequence-specific DNA binding and methylation activities.	Complex consisting of two restriction and two modification subunits.	Methylation and DNA cleavage reactions occur simultaneously. Translocation of DNA is driven by ATP hydrolysis. DNA cleavage occurs at a fixed distance on one side of the recognition site, and only when unmethylated recognition sites are inversely oriented. Methylation has no specific requirements as to the number and orientation of sites.
IV	Not relevant.	Complex is variable, containing one (Mrr, McrA) or two (McrBC) kinds of subunits.	Double-stranded cleavage of modified DNA is preceded by GTP hydrolysis-driven DNA translocation, and occurs at sites away from the recognition sequence.

*Bacillus anthracis* was discovered by Robert Koch in 1875. However, our knowledge of various aspects of its biology is far from complete. In the wake of advances in genetic engineering, attempts to transform *B. anthracis* with DNA indicated that it could be transformed only with DNA that had been obtained from *B. subtilis* strain 168 or the methylation-deficient *E. coli* strains JM110 and SCS110 (Marrero and Welkos, 1995). These observations highlighted the lack of systematic information on R–M systems in *B. anthracis*.

## R–M Systems in *B. anthracis*

Several strains of *B. anthracis* have been sequenced, their genomes analyzed for the presence of restriction enzymes, and the results compiled in the restriction enzyme database, REBASE (Roberts et al., 2015). Interestingly, most of these strains have identical profiles insofar as R–M systems are concerned. Searching REBASE (http://rebase.neb.com) reveals that the sequenced strains also apparently lack complete R–M systems of types I-III, and that virulence plasmids pXO1 and not encode any identifiable R–M systems either. There is a single ‘orphan’ type II DNA MTase (BA_3814) that is borne on prophage LambdaBa01 (Sozhamannan et al., 2006) and present in most sequenced strains, as revealed by a search of REBASE. There are at least four genes that encode components of type IV MDREs – MrrP (BA_2317), McrBP (BA_0927), McrB2P (BA_0928), and McrB3P (BA_2283) – with reference to the virulent Ames strain (Sitaraman and Leppla, 2012). This uniformity again attests to the observed monophyletic nature of *B. anthracis* as compared to closely related species such as *B. cereus* and *B. thuringiensis* (Helgason et al., 2000). The nomenclature used here follows from that of *E. coli* K-12 wherein two kinds of MDREs are known – Mrr (methylated-adenine recognition and restriction) that degrades not only adenine- but also cytosine-methylated DNA, and McrA and McrBC (methyl cytosine restriction) that specifically degrade methylated cytosine residues (Casali, 2003). It must also be noted that the virulence plasmids of *B. anthracis* (pXO1 and pXO2) do not encode any recognizable components of R–M systems in the strains where their sequences are available (Okinaka et al., 1999; Pannucci et al., 2002).

### Some Observations on Type IV Restriction Enzymes/MDREs in *B. anthracis* and Its Relatives

As stated above, most of the sequenced strains of *B. anthracis* encode the same kind and number of type IV MDREs. However, the Mrr enzyme exhibits significant variations, with the A2012 and Sterne strains exhibiting N-terminal deletions of 51 and 29 amino acids, respectively, relative to the Ames strain. The McrB2P protein exhibits a minor deletion of one N-terminal amino acid in strains A0248, A2012, and CDC684. Notably, the Sterne strain exhibits a large number of amino-acid deletions in the MDREs relative to the Ames strain (see Table 4 in Sitaraman and Leppla, 2012), but still retains the same complement of MDREs. One difference between the Mrr- and McrBC-type enzymes is that Mrr functions independently, whereas the Mcr enzymes function as complexes of McrB and McrC subunits. Of the Mcr polypeptides in the holoenzyme, McrB harbors DNA recognition and GTP hydrolysis activities (Pieper et al., 1999). McrC stimulates the GTPase activity of the McrB subunit and catalyzes DNA cleavage (Pieper and Pingoud, 2002). In *B. anthracis*, McrB2P exhibits a characteristic PD-X_n_-(D/E)XK nuclease/phosphodiesterase motif in common with known McrC subunits. (Therefore, the prevalent designation is somewhat confusing, because the McrC subunit is currently termed ‘McrB2P.’) By contrast, McrBP and McrB3P both contain a GTPase/ATPase domain. This implies that two different McrBC enzymes of differing specificity may be generated by complexes of McrB2P (the *B. anthracis* McrC) with either of the McrB homologs – McrBP McrB3P.

Examination of REBASE indicates that the genomes of both *B. cereus* and *B. thuringiensis* encode type IV restriction enzymes that are highly homologous to those encoded by *B. anthracis. B. cereus* and *B. thuringiensis*, in contrast to *B. anthracis*, exhibit considerable interstrain diversity in terms of the MDREs encoded. A partial listing of the closest homologs to the *B. anthracis* MDREs in *B. cereus* and *B. thuringiensis* is compiled in **Table 2**. Homologs of the *B. anthracis* McrB3P protein are the most ubiquitous in the *B. cereus* group, and those of Mrr the least. *B. thuringiensis* serovars Konkukian and Al Hakam contain two homologs each of *B. anthracis* MDREs. *B. cereus* strains Q1 and B4264 encode McrBP, McrB2P, and McrB3P. The maximum percentage of identity and similarity (computed by BLAST) at the DNA and protein levels in both these strains of *B. cereus*, as well as the Al Hakam and konkukian strains of *B. thuringiensis* to the *B. anthracis* MDRE genes and proteins is given in **Table 2**. Note that *mcrBP* and *mcrB2P* occur as part of an operon in *B. anthracis*, and the linkage is conserved in the listed *B. cereus* strains, with the exception of strain ATCC 10987. The high level of identity observed at the DNA level among homologs in these three species underscores the close phylogenetic relationship between them.

**Table 2:** Homologs of *Bacillus anthracis* MDREs in *B. cereus* and *B. thuringiensis* strains.

*B. anthracis* Ames MDRE as given in REBASE, Protein ID	*B. cereus*/*B. thuringiensis* strains containing homologs	REBASE protein designation	Protein ID	Remarks
BatAMrrP PI:AAP26188.1	B.c AH820	Bce820MrrP	ACK91564.1	99% identical (DNA and protein)
	B.t. serovar konkukian str. 97-27	BthKMrrP	AAT59812.1	99% identical (DNA); 98% identical (protein)
BatAMcrBP PI:AAP24919.1	B.c. AH187	BceAHMcrBP	ACJ82488.1	94% identical (DNA); 93% identical (protein)
	B.c. Q1	BceQMcrB1P	ACM11436.1	93% identical (DNA and protein);
	B.c. ATCC 10987	BceSMcrBP	Not available	Frameshift(s) in gene sequence
	B.c. B4264	BceB4264McrB2P	ACK59757.1	85% identical (DNA); 82 % identical (protein)
	B.t. Al Hakam	BthAHMcrBP	ABK84210.1	92% identical (DNA); 93% identical (protein);
BatAMcrB2P PI:AAP24920.1	B.c. Q1	BceQMcrB2P	ACM11437.1	93% identical (DNA); 95% identical (protein)
	B.c. B4264	BceB4264McrB3P	ACK59160.1	92% identical (DNA); 95% identical (protein)
	B.c. AH187	BceAHMcrB2P	ACJ81648.1	93% identical (DNA); 96% identical (protein)
	B.t. Al Hakam	BthAHMcrB2P	ABK84211.1	92% identical (DNA); 95% identical (protein)
BatMcrB3P PI:AAP26155.1	B.c. AH820	Bce820McrB2P	ACK88007.1	99% identical (DNA and protein)
	B.c. 03BB102	Bce03McrBP	ACO30889.1	99% identical (DNA and protein)
	B.c. ATCC 10987	BceSMcrB2P	AAS41236.1	97% identical (DNA);98% identical (protein)
	B.c. Q1	BceQMcrB3P	ACM12648.1	97% identical (DNA and protein)
	B.c. G9842	BceGMcrBP	ACK95211.1	89% identical (DNA and protein)
	B.c.B4264	BceB4264McrB4P	ACK63998.1	88% identical (DNA); 89% identical (protein)
	B.c. ATCC 14579	Bce14579McrBP	Not available	Frameshift(s) in gene sequence
	B.c. ssp. *cytotoxis* NVH 391-98	Bce98McrBP	ABS21980.1	76% identical (DNA and protein)
	B.t. serovar konkukian str. 97-27	BthKMcrBP	AAT59793.1	99% identical (DNA and protein)

*B. cereus and *B. thuringiensis* strains having homologs to the largest number of *B. anthracis* (Ames strain) MDREs are underlined. Percentage identities at the DNA and protein levels were computed using BLASTN and BLASTP online tools, respectively, (http://blast.ncbi.nlm.nih.gov/). B.c. – *Bacillus cereus*; B.t. – Bacillus thuringiensis.*

As stated earlier, transformation of *B. anthracis* requires unmethylated DNA, suggesting the presence of one or more active MDREs. Transformation experiments in *B. anthracis* strain UM44-1C9 (a plasmid-free, avirulent Sterne-type strain) mutated in one or more of the identified type IV MDRE-encoding loci indicate that all these enzymes may be active against C-5 and/or N6-A methylated DNA. Evidence from such experiments as well as information in microarray expression databases indicates that these MDRE loci are transcribed at varying levels, and the enzymes so expressed likely contribute to the degradation of C5-methylated DNA (especially CpG-methylated DNA), and with differing efficiencies (Sitaraman and Leppla, 2012).

### The Prophage-Borne Orphan DNA MTase (BA_3814) of *B. anthracis*

The *B. anthracis* genome harbors four excision-proficient prophages, of which LambdaBa01 encodes a putative type II C-5 DNA MTase that does not seem to be paired with a cognate RE (Sozhamannan et al., 2006). Its recognition sequence is predicted to be the same as M.EaeI (5′-(C or T)GGCC(A or G))-3′ or M.EagI (5′-CGGCCG-3′), with C-5 methylation occurring on the innermost C residue. Note that the four central nucleotides of both predicted sequences are identical to the M.HaeIII recognition site (5′-GGCC-3′). We note that the *B. anthracis* Ames genome contains 163 EagI and 1064 EaeI sites making methylation, if it occurs, quite frequent. Currently, we have strong indications that the *B. anthracis* MDRs cleave cytosine-methylated DNA sequences, because wild-type *B. anthracis* UM44-1C9 can be transformed with unmethylated plasmid DNA, but not when the same same plasmid DNA is methylated by M.HaeIII *in vitro* (Sitaraman and Leppla, 2012). Therefore, it is quite surprising that the orphan DNA MTase BA_3814 with a potential M.HaeIII-like sequence specificity has been acquired and is stably carried on the prophage in practically all sequenced strains of *B. anthracis* and some strains of *B. cereus*, indicating that it has to be inactive or at least reliably prevented from methylating the host genomic DNA. The other formal possibility is that the DNA sequence specificity predicted for BA_3814 is incorrect.

### A Type II Restriction Enzyme in *B. anthracis*?

Although genome sequencing has indicated that *B. anthracis* strains uniformly lack type II enzymes, there has been a single report of the identification of a type II restriction enzyme (designated BanAI, REBASE number 6143) in a strain of *B. anthracis* isolated from the Amazon basin (Chies et al., 2002). This enzyme was found to be an isoschizomer of HaeIII, recognizing and cleaving the sequence 5′-GG|CC-3′, but both its aminoacid and gene sequences remain unknown. As pointed out in the previous section, HaeIII-type methylation of the *B. anthracis* genome would likely render it susceptible to attack by the resident MDREs. This implies that either MDREs are absent or inactive in the strain of *B. anthracis* studied by Chies et al. (2002), or there could be some uncertainty regarding the species identification in their study. Data accumulating since 2002 indicate that the latter case is more likely to be the correct explanation. In fact, while there are no BanAI isochizomers or even type II REs encoded by any known strains of *B. anthracis*, a search of REBASE indicates these are present in three strains of *B. cereus* (BceWORF97P – strain W; Bce1407ORF2765P – strain 1407; Bce71I – strain 71). Of these, the first two are predicted, and the last is characterized, but not commercially available.

At the time, the 16S rRNA coding sequences of the Amazon isolate (GenBank Acc. No. AY043083.1) exhibited 100% identity with *B. anthracis* (Ames strain), but genome sequencing has failed to uncover any gene that might encode a type II restriction endonclease. Re-analysis of the 16S rDNA sequences by a simple megaBLAST search against bacterial and archaeal genomes indicates that sequences of *B. anthracis* strain ATCC14578, and *B. cereus* strains CCM 2010, IAM 12605, ATCC 14579, NBRC 15305, and JCM 2152, exhibit 99% nucleotide identity with that of the Amazon isolate. Therefore, given the close similarity between members of the *Bacillus* group, it is likely that the Amazon isolate might also be a strain of *B. cereus* or another closely related *Bacillus* sp. This isolate must necessarily possess the cognate DNA MTase methylating HaeIII sequences to shield its own genome, and might offer valuable clues to the dispersal of BA_3814 within the *Bacillus* group, especially if the latter is also found to methylate HaeIII sequences.

## Concluding Remarks

What is the role of multiple type IV restriction enzymes in the biology of a soil-dwelling, zoönotic pathogen? In general, restriction enzymes can confer protection against attack by bacteriophages. However, lysogenization by phages in the soil may also have an adaptive value for *B. anthracis* (and perhaps other bacteria) under natural conditions, altering sporulation, exopolysaccharide production, biofilm formation, and even promoting colonization of earthworms as intermediate hosts via phage-encoded sigma factor-mediated activation of specific *B. anthracis* genes (Schuch and Fischetti, 2009). Among bacteria, transduction by phages as well as incursions of mobile genetic elements via conjugation contributes to horizontal gene transfer (HGT). Orphan DNA MTases encoded by phages, while protecting phage DNA from host R–M systems (Murphy et al., 2013), could conceivably contribute to gene regulation of host genes by methylation of regulatory sequences when integrated for the long term into the host genome as prophages.

As stated before, it is well-recognized that *B. cereus, B. thuringiensis* and *B. anthracis* are very closely related, and the data in **Table 2** corroborates this inference. Examination of REBASE reveals that the MDREs in *B. anthracis* are only a subset of those observed in *B. cereus* and *B. thuringiensis* that possess MDREs in greater numbers and diversity. Additionally all *B. anthracis* strains in REBASE encode the identical set of R–M genes (though with polymorphisms as noted earlier), a fact of potential importance in the context of monophyly in the *B. anthracis* lineage, besides potentially serving as a marker for this lineage. The prior presence of a type IV R–M system in a cell can prevent infection by phages or acquisition of mobile genetic elements if their DNA is modified, thereby limiting their spread and the scope for HGT. It has been demonstrated in *E. coli* K-12 that the resident McrBC antagonizes the establishment of type II R–M genes encoding PvuII (from *Proteus vulgaris*) by destroying methylated host genomic DNA that results from PvuII acquisition (Fukuda et al., 2008), thereby overriding the potentially ‘selfish’ proclivities of the intruding type II R–M system. Similarly, the *E. coli* Mrr antagonizes the establishment of the type III StyLTI R–M system derived from *Salmonella typhimurium* LT2 in *E. coli* MG1655, and proves toxic when introduced into *S. typhimurim* LT2 (Tesfazgi Mebrhatu et al., 2011). However, more extensive analysis demonstrated that the presence of McrA and McrB in *E. coli* K-12 prevented the cloning of type II MTases only of certain specificities (Raleigh and Wilson, 1986). Also note that *E. coli* K-12 encodes EcoKI (a type I R–M system) and two DNA MTases (Dam and Dcm) on the one hand, and three known MDREs – Mrr, McrA and McrBC, on the other – all without apparent conflict (Casali, 2003). Likewise, *B. cereus* AH187 possesses close homologs of *B. anthracis* MrrP, McrBP, and McrB2P (see **Table 2**), and also a putative type III R–M system. Interestingly, most strains of *Helicobacter pylori*, a gastric pathogen, routinely encode large numbers (∼20–25) of putative type II MTases, as well as type I and type III R–M systems, but are generally found to be deficient in functional type IV restriction enzymes. For example, strain Aklavik86 encodes several type I, II, and III R–M systems, but has only an McrB-type subunit with no recognizable McrC. The foregoing observations indicate that MDREs do not necessarily conflict with the acquisition of the other three types of R–M systems provided their DNA sequence specificities do not overlap. A corollary is that, increasing genome methylation by several resident DNA MTases reduces the likelihood of having or acquiring one or more functional MDREs.

The interactions between resident, incoming and neighboring R–M systems, as well as the phages in the environment have implications for HGT. Recent mathematical modeling of the impact of R–M systems (implicitly assumed to be types I–III) on bacterial diversity when infected by a single phage type indicates that their ability to methylate, rather than restrict, a fraction of infecting phage DNA facilitate the long-term coexistence of multiple strains by preventing any one strain from dominating the community (Sneppen et al., 2015). In terms of specificity of DNA sequence recognition, relaxed specificity of wild-type KpnI (a type II RE) expressed in *E. coli* has been found to confer a fitness advantage to the host relative to one harboring a high-fidelity version of the same enzyme (Vasu et al., 2012) However, it remains to be seen what impact the *B. anthracis* MDREs have on genetic diversity, and whether they are responsible in some manner for the minimal interstrain variation observed in *B. anthracis.* It is conceivable that relaxed sequence specificity would lead to more effective exclusion of not only phage but also other types of DNA that happen to be modified owing to prior carriage in other bacteria that encode one or more DNA MTases. Thus, if *B. anthracis* were to invade a bacterial community consisting predominantly of methylation-competent bacteria, it might be more capable of withstanding phage attack and be susceptible only to a minority of phages that either do not contain methylated DNA or fortuitously contain methylated DNA that is compatible with *B. anthracis* MDREs. Given the ubiquity of R–M systems among prokaryotes, this scenario is quite probable. Finally, degradation of CpG-methylated mammalian host DNA during pathogenesis, or that of methylated DNA in the free-living state by the MDREs would increase the availability of free nucleotides for *B. anthracis.*

Methylation-dependent restriction enzymes are not ‘selfish’ in the sense paired R–M systems of the other three types are. Therefore, while they do prevent the acquisition of new R–M systems of types I–III, or methylated DNA, their loss (or lack of expression) will not result in post-segregational killing of the host cell, as mutants are readily obtainable (Sitaraman and Leppla, 2012). Thus, phase variation in MDRE expression might permit bacteria to alternate receptive and refractory phases, especially in naturally transformable species, but there are no documented instances of such behavior in *B. anthracis* or other members of the *B. cereus* group. The ComK protein, the master regulator of competence in *B. subtilis*, has been detected in several *B. cereus* strains (Mirończuk et al., 2008) and ComK homologs as well homologs of several competence-related structural proteins are also present in *B. anthracis* (strains Ames, Ames0581, and Sterne), indicating that the monophyletic nature of this bacterium may not be due to a lack of competence machinery (Kovács et al., 2009). By limiting HGT, R–M systems could help maintain bacterial species identity (Jeltsch, 2003). Interestingly, R–M system combinations can help distinguish strains of *Xylella fastidiosa* (Van Sluys et al., 2003), and regroup *Neisseria meningitides* strains into ‘phylogenetic clades’(Budroni et al., 2011). Thus, while MDREs are convenient markers for the *B. anthracis* lineage, their roles in its intraspecific uniformity and ecological success merit further investigation.

## Conflict of Interest Statement

The author declares that the research was conducted in the absence of any commercial or financial relationships that could be construed as a potential conflict of interest.
